# Lipidic Nanoparticles, Extracellular Vesicles and Hybrid Platforms as Advanced Medicinal Products: Future Therapeutic Prospects for Neurodegenerative Diseases

**DOI:** 10.3390/pharmaceutics16030350

**Published:** 2024-03-01

**Authors:** Maria Tsakiri, Ioannis Tsichlis, Cristina Zivko, Costas Demetzos, Vasiliki Mahairaki

**Affiliations:** 1 Section of Pharmaceutical Technology, Department of Pharmacy, School of Health Sciences, National and Kapodistrian University of Athens, Panepistimioupolis Zografou, 15771 Athens, Greece; tsakirim@pharm.uoa.gr (M.T.); gtsichlis@pharm.uoa.gr (I.T.); demetzos@pharm.uoa.gr (C.D.); 2 Department of Genetic Medicine, Johns Hopkins School of Medicine, Baltimore, MD 21287, USA; czivko1@jhmi.edu; 3 The Richman Family Precision Medicine Center of Excellence in Alzheimer’s Disease, Johns Hopkins University School of Medicine, Baltimore, MD 21287, USA

**Keywords:** lipid-based nanoparticles, liposomes, extracellular vesicles, hybrid liposome–EVs, neurodegeneration

## Abstract

Neurodegenerative diseases, such as Alzheimer’s and Parkinson’s, affect a wide variety of the population and pose significant challenges with progressive and irreversible neural cell loss. The limitations of brain-targeting therapies and the unclear molecular mechanisms driving neurodegeneration hamper the possibility of developing successful treatment options. Thus, nanoscale drug delivery platforms offer a promising solution. This paper explores and compares lipidic nanoparticles, extracellular vesicles (EVs), and hybrid liposomal–EV nanoplatforms as advanced approaches for targeted delivery to combat neurodegeneration. Lipidic nanoparticles are well-characterized platforms that allow multi-drug loading and scalable production. Conversely, EVs offer the ability of selectively targeting specific tissues and high biocompatibility. The combination of these two platforms in one could lead to promising results in the treatment of neurodegeneration. However, many issues, such as the regulatory framework, remain to be solved before these novel products are translated into clinical practice.

## 1. Introduction

Neurodegeneration includes several disorders characterized by the progressive loss of function of the neural cells in the brain or peripheral nervous system. The most common neurodegenerative diseases include Alzheimer’s disease, Parkinson’s disease, Huntington’s disease, and multiple sclerosis [1]. 

Alzheimer’s disease (AD) has a great impact on both the patient and their social environment. More specifically, AD is the main cause of dementia in elderly people and is characterized by a progressive decrease in memory and cognitive function [2]. The key pathophysiology of AD includes the progressive loss of neurons, the accumulation of extracellular beta-amyloid (Aβ) peptides, and intracellular neurofibrillary tangles. Also, the activation of microglia and astrocytes triggers the release of proinflammatory mediators, including cytokines and chemokines, leading to chronic inflammation and neuronal dysfunction. The blood–brain barrier (BBB) breakdown allows the infiltration of immune cells, leading to a prolonged inflammatory response and therefore neurodegeneration [3]. However, the molecular mechanisms of neurodegeneration in AD remain to be fully elucidated [1].

Parkinson’s disease (PD) is the second most common neurodegenerative disorder, and it is characterized by motor symptoms, such as akinesia, bradykinesia, tremors, and rigidity [4]. Patients may show additional non-motor symptoms, like sleep disorders, psychiatric disorders, and sensory disturbances [5]. The pathophysiology of PD includes synaptic transport abnormalities, mitochondrial dysfunction, and neuroinflammation [3]. Specifically, the destruction of dopaminergic neurons in the substantia nigra and the presence of Lewy bodies, abnormal protein aggregates that include alpha-synuclein and ubiquitin, contribute to disease progression [4]. Alpha-synuclein is a small amyloid protein that is abundant in the brain, mainly at the presynaptic terminals. Lewy bodies can disrupt the concentration of dopamine to toxic levels, leading to neuronal death [6,7]. Researchers also suspect that Lewy bodies stop or disrupt the cellular mechanism that removes proteins that are not needed. Thus, neurodegenerative disorders pose a significant challenge, as current treatments are only able to alleviate a few symptoms or slow the inevitable progression of these diseases [8].

In addition to the complexity of the underlying pathophysiology in neurodegeneration, a major obstacle is effective drug delivery in the central nervous system (CNS). The BBB is a metabolic and physical barrier with tight junctions that regulate and limit the entry of bioactive molecules into the brain. While a lot of research has been conducted on ways to enhance the permeability and accumulation of therapeutic molecules through the BBB, many different novel strategies are being developed to bypass the BBB. For instance, nasal drug delivery presents encouraging results [9,10]. By intranasal delivery, the drugs can pass directly to the CNS via the olfactory and trigeminal nerves. Both parenteral and nasal administration delivery platforms aid the accumulation of therapeutic molecules in the CNS [11,12]. 

Nanoscale active pharmaceutical ingredient (API) delivery platforms have emerged as promising candidates for targeted drug delivery to the brain due to their ability to be designed with specific physicochemical properties. They can pass through the BBB based on size, ζ-potential, morphology, surface modifications, and lipophilicity. These characteristics can impact their pharmacokinetics for optimal drug delivery and enhanced targeting efficiency [13]. Moreover, they provide both physicochemical and biological protection of the API after in vivo administration and enable targeted API release [14,15]. In this way, they enhance therapeutic efficiency while mitigating systemic adverse effects [16].

Today, alongside synthetic nanoplatforms such as lipid, polymeric, and inorganic nanoparticles, the inclusion of biologically derived vehicles has expanded the spectrum of nanotechnological approaches [17]. Although the main role of extracellular vesicles (EVs) in medical applications revolves around early diagnosis [17,18], recent studies have shown their potential benefits as advanced API delivery nanoplatforms [19,20]. 

This paper aims to explore key API delivery nanoplatforms employed to alleviate neurodegeneration. Our objective is to present an overview of recent advancements in lipidic nanoparticles for the efficient transport of therapeutic molecules to delay the progress of neurodegenerative disorders. Subsequently, we discuss research conducted on the utilization of EVs for the same therapeutic objectives. Lastly, we present recent advances in the field of hybrid liposomal–EV nanoplatforms, raising the question of their prospective advantages in tackling neurodegeneration. In this review, we aim to provide a critical overview of the evolving landscape of strategies and technologies for delaying the progression of neurodegenerative disorders.

## 2. Lipidic Nanoparticles against Neurodegeneration

**Figure 1:**
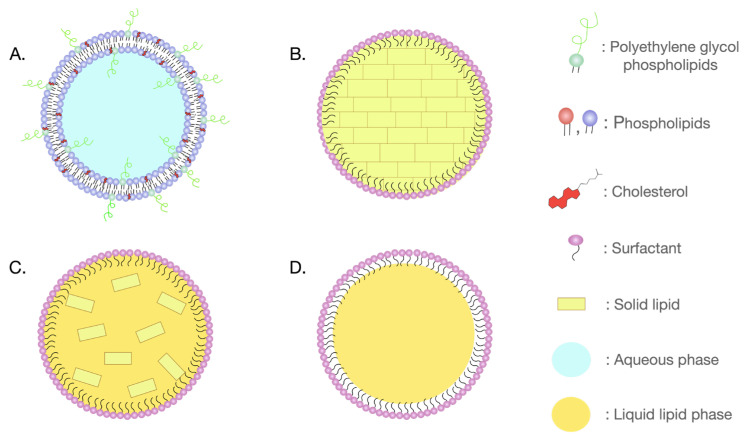
Subcategories of lipidic nanoparticles: (**A**) liposome; (**B**) solid lipid nanoparticle (SLN); (**C**) nanostructured lipid carrier (NLC); and (**D**) nanoemulsions.

Lipidic nanoparticles, including liposomes, solid lipid nanoparticles (SLNs), nanostructured lipid carriers (NLCs), nanoemulsions, and lipid nanocapsules (LNCs) can offer several advantages in drug delivery (Figure 1). Liposomes, spherical vesicles composed of one or more lipid bilayers, are biocompatible and can entrap hydrophilic compounds within the aqueous core and lipophilic compounds into their lipid bilayers. Moreover, they can be modified with specific molecules/ligands for tissue-specific drug delivery while minimizing the accumulation of drugs in healthy tissues and limiting toxic, off-target effects [21]. SLNs, composed entirely of solid lipids and stabilized by surfactants, are biocompatible and can effectively deliver several molecules. On the other hand, NLCs, blending solid and liquid lipids in a partially crystallized matrix and surfactants in the aqueous phase, excel in enhancing drug encapsulation and preventing leakage during storage [22,23]. LNCs are characterized by a core–shell structure. The oily core typically contains the drug and is surrounded by an external shell composed of solid lipids and surfactants. These nanoparticles are biocompatible and exhibit superior colloidal stability [24]. Nanoemulsions are composed of oil droplets dispersed in an aqueous medium and they are stabilized by surfactants. They possess several advantages including improved bioavailability, drug loading, and targeted drug delivery [25]. All the above lipid-based nanoparticles can be used as effective vehicles for delivering drugs to the brain, with potential for controlled release and improved bioavailability [26].

Several techniques have been utilized to bypass the BBB, including invasive methods such as injection into the cerebrospinal fluid (CSF) and intracerebroventricular injection. However, neurosurgery poses high risks and has limited efficacy [27]. Nanoparticles, including lipid-based ones, can effectively enter the brain through the disruption of tight junctions and transcytosis, which is mediated by low-density lipoproteins, insulin, and transferrin receptors [28,29]. Lipid-based nanoparticles, upon entering the brain, interact with the microglial cell membrane through endocytosis. This process involves phagocytosis for larger particles and pinocytosis for smaller ones, which are further classified into clathrin-mediated, caveolae-mediated, clathrin- and caveolae-independent, and micropinocytosis processes [30]. In addition to these active transport mechanisms, nanoparticles can also penetrate cells through passive diffusion. Their physicochemical properties can significantly affect microglial modulation. Specifically, nanoparticle size and surface modifications can influence their retention time and subsequently the concentration of nanoparticles within microglia [31].

Lipid-based nanoparticles exposed to biological fluids interact with plasma components, leading to the formation of a protein corona. The composition of this corona is also influenced by the physicochemical characteristics (i.e., lipid composition, size, ζ-potential, surface modifications) of the nanoparticles and depends on the biological environment in which they are dispersed. This modulation affects the physiological interactions of the nanoparticles in vivo, influencing pharmacokinetics and biodistribution, and targeting capability. However, the protein corona can also be utilized as a strategy to target nanoparticles to specific tissues [32].

Different researchers have utilized small molecules, including antioxidant compounds, peptides, and antibodies, for inhibiting pathological protein aggregation. Also, several lipidic nanoparticles have been developed to target neurodegeneration associated with AD (Table 1). Specifically, dual-modified liposomes with ApoE-derived peptides for BBB targeting and phosphatidic acid for Aβ binding have demonstrated promising potential in addressing AD pathology. Research studies involving APP/PS1 and APP23 transgenic mice highlight the effectiveness of this formulation in reducing amyloid load. This was indicated by a decrease in both plasma and brain Aβ levels [33]. Furthermore, liposomes loaded with the antioxidants quercetin and rosmarinic acid, incorporating apolipoprotein E and phosphatidic acid, have been successful in repairing Aβ1-42-induced neurotoxicity in AD due to enhanced BBB penetration. This liposomal formulation exhibited neuroprotective effects by decreasing acetylcholinesterase activity, lipid peroxidation in the hippocampus, and Aβ plaque formation in the brain of AD rat models [34]. 

Oxidative stress, particularly due to lipid peroxidation in the brain, has long been linked to AD neurodegeneration [35]. Several clinical trials have been evaluating the use of the antioxidant curcumin in AD [36], but its hydrophobicity often results in poor bioavailability [37]. To overcome this challenge, lipid-core nanocapsules were loaded with curcumin, demonstrating substantial efficiency leading to significant reductions in neuroinflammation, behavioral impairments, and the hyperphosphorylation of tau and Aβ in a murine model of AD [38]. Resveratrol, a natural polyphenol found in various plants, has also gained attention for its potential therapeutic effects in AD. Its powerful antioxidant properties could play a crucial role in addressing oxidative stress implicated in AD progression. However, the hydrophobic nature of resveratrol limits its use due to low solubility and bioavailability. Therefore, several lipidic nanoparticle formulations incorporating resveratrol have been studied to overcome these limitations and improve AD therapeutics. In a recent study, liposomes modified with ApoE incorporating resveratrol and salidroside were proven effective in bypassing the BBB in vitro and exhibited neuroprotective effects in APP/PS1 transgenic mice [39]. Another research study demonstrated that resveratrol-loaded lipid-core nanocapsules reduced neuroinflammation in a rat organotropic hippocampal culture exposed to Aβ1–42 and alleviated Aβ1–42 harmful effects. This was indicated by a reduction in proinflammatory cytokines (i.e., TNF-a, IL-1β, IL-6) along with a decrease in both glial and c-Jun N-terminal kinase activation [40]. In another study, lipidic nanoparticles loaded with epigallocatechin gallate, another polyphenolic compound derived from plant extracts, showed enhanced bioavailability and a-secretase activity in mouse models of AD, demonstrating potential therapeutic outcomes [41]. 

Lipidic nanoparticles have also been utilized to deliver therapeutic molecules to the CNS by modifying their surface with specific ligands such as transferrin and lactoferrin. In 2020, Kon et al. developed transferrin-modified PEGylated liposomes to enhance the BBB penetration of a coumarin compound called osthole that stimulates neural stem cells and reduces Aβ peptides. The results showed that this formulation successfully improved the pharmacodynamics and pharmacokinetics of osthole, reducing amyloid beta in APP/PS1 mice and therefore indicating that it could be a potential strategy for addressing AD [42]. Another research group developed quercetin-loaded liposomes modified with lactoferrin and a bradykinin analog called RMP-7. The results showed that these surface modifications enhanced the BBB penetration of quercetin and notably diminished Aβ-induced neurotoxicity in vitro [43]. Overall, these formulations highlight the potential of lipidic nanoparticles not only in Aβ reduction, but also in delivering therapeutic molecules for fighting neuroinflammation and associated pathologies in AD [40]. 

Several studies are focused on eliminating the oxidative stress mechanisms observed in PD as well. Therefore, potent antioxidant compounds are utilized to alleviate PD symptoms. Pangeni et al. developed a resveratrol-loaded vitamin E nanoemulsion to target and mitigate PD symptoms. The results demonstrated that the intranasal administration of the nanoemulsion led to an elevated concentration of antioxidants in the brain. Subsequently, histopathological analysis in rats’ brains indicated a significant reduction in degeneration [44]. In addition, another study highlights the potential use of liposomal glutathione (GSH) for maintaining intracellular GSH levels in neuronal cells. This formulation exhibited enhanced protection to neurons in a Parkinson’s in vitro disease model using rat mesencephalic cells [45]. 

Despite the potential and promising role of antioxidative and anti-inflammatory molecules in preventing neurodegenerative disorders, there is significant discussion over their ability to repair damaged neurons, organelles, and proteins [46]. Nowadays, research is focused on targeting microglia to address neurodegeneration, as these immune cells are involved in protein trafficking, aggregation, and clearance, and have a pivotal role in neuroinflammation. In a recent study, liposomes modified with glycan ligands of CD33 showed increased phagocytosis by microglia in a CD33-dependent manner. The researchers found that this effect is associated with the loss of CD33 from the cell surface and the internalization of liposomes. Notably, this study demonstrates that the multivalent engagement of CD33 with glycan ligands can modulate microglial cell function, offering a promising strategy for treating neurodegeneration, particularly AD [47]. Tentillier et al. investigated the potential of CD163-targeted glucocorticoids to protect dopaminergic neurons in a 6-hydroxydopamine PD model in rats. The authors found that CD163-targeted liposomes loaded with dexamethasone were able to selectively deliver the drug to CD163 macrophages in the brain, leading to improved motor performance and increased striatal TH+ fiber innervation compared to the control animals. These results suggest that the anti-inflammatory modulation of microglia via CD163-targeted glucocorticoids may be a promising therapeutic strategy for Parkinson’s disease [48]. Another recent study demonstrates that treatment with brain-targeted liposomes loaded with SynO4 monoclonal antibodies inhibits alpha-synuclein aggregation. This treatment consequently reduced the activation of microglia cells and the associated neuroinflammation in PD mice [49]. 

Based on the above studies, we can conclude that the composition of the lipidic nanocarrier not only influences the physicochemical characteristics of the nanocarrier, but can also significantly affect drug delivery to the brain. More precisely, surface modification of the nanocarrier with specific ligands that interact with receptors or transporters of the brain can facilitate transcytosis and enhance BBB penetration. Also, the addition of PEGylated lipids can increase circulation time and, therefore, reduce clearance by the reticuloendothelial system and enhance brain delivery. Different types of biocompatible lipidic nanoparticles are being used to overcome a variety of aspects affecting treatment possibilities in neurodegenerative diseases such as AD and PD. The versatility of these artificial drug delivery platforms could be used to improve the bioavailability of water-insoluble compounds, while their physicochemical properties can be fine-tuned to enhance BBB crossing.

**Table 1:** Studies utilizing lipidic nanoparticles and extracellular vesicles as delivery nanosystems.

Disease	Composition /Source	Method of Development /Isolation	Therapeutic Cargo	Targeting Factors	Evaluation Model	Main Findings	Ref.
**Lipidic Nanoparticles**							
AD	Sphingomyelin, cholesterol, phosphatidic acid	Thin-film hydration/Extrusion	-	Phosphatidic acid, mApoE	APP/PS1 transgenic mice APP23 transgenic mice	- Decrease in Aβ levels, plaque reduction, ameliorated memory impairment.	[33]
AD	DPPC, DHDP, DSPE-PEG, cholesterol, phosphatidic acid	Thin-film hydration/Extrusion	Quercetin, Rosmarinic acid	Phosphatidic acid, ApoE	HBMEC/HA/ HBVP cells SK-N-MC cells AD rat model (Aβ1–42)	- Decrease in acetylcholinesterase activity and lipid peroxidation level, and lower Aβ plaque formation.	[34]
AD	Poly(ε-caprolactone), capric/caprylic triglycerides, sorbitan monostearate, Tween 80	Nanoprecipitation	Curcumin	-	AD mouse model (Aβ_25–35_)	- Antidepressant-like and antioxidant effects.	[38]
AD	Lecithin, cholesterol, DSPE-PEG, DSPE-PEG-NHS	Thin-film hydration/Sonication	Resveratrol Salidroside	ApoE	APP/PS1 mice bEnd.3 mouse brain cell line N2a cell line	- Increased levels of SOD and GSH-Px. - Decreased levels of MDA. - Decrease in TNF-a, IL-1β, and IL-6. - Decrease in Iba-1 in microglia and GFAP in reactive astrocytes. - Inhibition of Bax expression. - Promotion of Bcl-2, BDND, and GDNF expression. - Improvement in learning and brain function.	[39]
AD	Poly(ε-caprolactone), capric/caprylic triglyceride, sorbitan monostearate, Tween 80	Nanoprecipitation	Resveratrol	-	Rat organotropic hippocampal culture exposed to Aβ_1–42_	- Inhibition of ROS formation, decrease in TNF-a, IL-1β, and IL-6, increase in IL-10, decreased glial and JNK activation.	[40]
AD	EPC, cholesterol, DSPE-PEG, DSPE-PEG-Maleimide	Thin-film hydration/Sonication	Osthole	Transferrin	hCMEC/D3/SH-SY5Y cell co-culture BBB model APP/PS1 mice	- Decrease in MDA. - Increase in SOD. - Decrease in TNF-a, IL-1β, IL-6, and Iba-1. - Decrease in Aβ1–42 levels and plaque deposition. - Improvement in learning and cognitive function.	[42]
AD	DPPC, SPC, cholesterol, stearylamine, cardiolipin, DSPE-PEG-CA	Thin-film hydration/Extrusion	Quercetin	Lactoferrin RMP-7	HBMEC/HA cells Aβ-insulted SK-N-MC cells	- Enhanced BBB penetration. - Decrease in Aβ-induced neurotoxicity. - Inhibited expression of phosphorylated c-Jun N terminal kinase, phosphorylated p38, and phosphorylated tau protein at serine 202.	[43]
PD	DSPC, cholesterol, DSPE-PEG, pHrodo-PEG-DSPE, CD33L-PEG-DSPE, AF647-PEG-DSPE	Thin-film hydration/Sonication/Extrusion	-	Glycan ligands of CD33	WT U937 cells CD33^−/−^ U937 cells HMC3 cells Primary human microglia cells Isolated microglia from hCD33M transgenic mice and WT mice	- Increased phagocytosis in a CD33-dependent manner.	[47]
PD	HSPC, cholesterol, mPEG2000-PE, lipidated CD163-antibody clone ED2	Ethanol injection/Extrusion	Dexamethasone	CD163	6-OHDA rat PD model	- Improved motor function and dopaminergic survival.	[48]
PD	DPPC, cholesterol, DSPE-PEG1000, DSPE-PEG2000-NH2 (or DSPE-PEG2000 for untargeted liposomes)	Thin-film hydration/Extrusion	SynO4 mAb	Transferrin	hCMEC/D3 cells	- Reduced a-synuclein aggregation and neuroinflammation. - Improvement in motor function and motor learning.	[49]
**Extracellular Vesicles**							
AD	Genetically modified dendritic cells	Ultracentrifugation	siRNA	RVG-peptide	Wild-type mice	- BACE-1 knockdown.	[50]
AD	Mesenchymal stem cells (MSCs)	Ultracentrifugation	MSC-derived EV cargo	RVG-peptide	APP/PS1 double transgenic mice	- Decrease in Aβ levels, plaque deposition and astrocyte activation. - Decrease in TNFα, IL-β, and IL-6.	[51]
PD	Murine dendritic cells	Ultracentrifugation	Anti-α-synuclein shRNA-minicircles	RVG-peptide	Normal and α-synuclein transgenic mice	- Reduced α-synuclein aggregation and dopaminergic neuron loss. - Improved clinical symptoms.	[52]
PD	HEK293T cell	Gradient centrifuge	DNA aptamers specific for α-synuclein	RVG-peptide	Wild-type mice injected with α-synuclein preformed fibril	- Effective delivery in mouse brain. - Reduction in α- synuclein aggregates. - Improved motor impairments.	[53]
NI	Murine macrophages (Raw 264.7 Mϕs)	Ultracentrifugation	BDNF	LFA-1	CD-1 mice	- Increased brain cellular uptake in neuroinflammation due to ICAM-1 upregulation.	[54]
AD	MSCs and Hypoxic progenitor MSCs	Polymer-based microspheres (ExoQuick-TC, System Bioscience, Palo Alto, CA, USA)	miR-21	-	APP/PS1 double transgenic mice	- Improved memory and learning capability. - Decreased Aβ level deposition, glial fibrillary acidic proteins, ionized calcium-binding adaptor molecule 1, TNF-α, and IL-1β. - Decreased activation of STAT3 and NF-κB. - Increased levels of growth-associated protein 43, synapsin 1, IL-10, and miR-21.	[55]
AD	MSCs	Polymer-based microspheres (ExoQuick-TC, System Bioscience, Palo Alto, CA, USA)	nk	-	APP/PS1 double transgenic mice	- Increased levels of pro-inflammatory cytokines. - Decreased levels of anti-inflammatory cytokines.	[56]
EAE	Adipose MSCs	Ultracentrifugation	nk	-	Chronic EAE C57Bl/6 mice	- Inhibition of T-cell extravasation in the inflamed CNS. - Reduction in T-cells adhesion to integrin ligands.	[57]
EAE	MSCs	Centrifugation, exosome isolation kit (Invitrogen, Waltham, MA, USA)	miR-467f, miR-466q	-	SOD1^G93A^ mice	- Decreased expression of neuroinflammation markers in the spinal cord of EAE-affected mice. - No effect on disease progression.	[58]
EAE	INF-γ activated MSCs	Ultracentrifugation	-	-	EAE C57BL/6J mice	- Reduction in demyelination and neuroinflammation. - Upregulation of Treg cells.	[59]

AD: Alzheimer’s disease; PD: Parkinson’s disease; NI: neuroinflammation; EAE: experimental autoimmune encephalomyelitis; INF: interferon; RVG: rabies virus glycoprotein; nk: not known.

## 3. Extracellular Vesicles against Neurodegeneration

**Figure 2:**
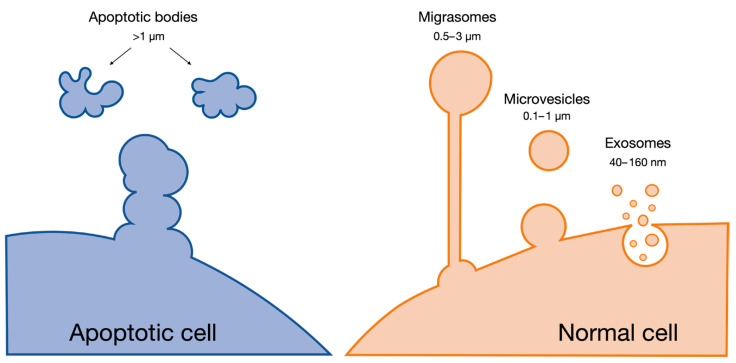
Main categories of extracellular vesicles.

Extracellular vesicles (EVs) are cell-derived nanoscale particles that are released in the extracellular milieu (Figure 2). EVs have an important role in both the intra- and inter-cellular communication of physiological and pathophysiological procedures [60]. Based on their biogenesis, size, and content, they can broadly be distinguished into three main categories: (i) exosomes, (ii) microvesicles, and (iii) apoptotic bodies [61]. Although there is some overlap between the categories, exosomes are formed through the endosomal pathway and released by multivesicular bodies, and have a size of 40–160 nm. Microvesicles and apoptotic bodies belong to the broad class of ectosomes; they are formed from plasma membranes and generally have size ranges of 100–1000 nm and above 1000 nm, respectively [62]. Migrasomes are another population of EVs that have recently been described. While the functional role of migrasomes is still under active investigation, emerging evidence suggests their involvement in cell migration, potentially by leaving tracks for other cells to follow or by serving as signaling entities during collective cell migration. Their size varies between 500 and 3000 nm and their travel range is limited, in contrast with other EVs such as exosomes [63,64].

The considerable overlap of some characteristics of the different types of EVs makes it difficult to separate them by classic biochemical procedures. Due to this, in 2018, the International Society of Extracellular Vesicles (ISEV) published updated guidelines that proposed the utilization of the terms small or large EVs instead of mentioning the exact EV type [65]. In the present review, we follow these guidelines when mentioning EVs.

The cargo and composition of EVs are diverse, reflecting the functionality of their parent cells and encompassing various molecules capable of activating distinct biochemical pathways. Facilitating cell-to-cell communication, EVs play a crucial role in maintaining homeostasis, identifying pathological activities, and signaling neighboring cells [60]. However, there are instances when EVs act as disease promoters, such as in the cases of cancer [66,67], neurodegeneration [68,69,70,71,72], and cardiovascular diseases [73,74]. 

The significant role of EVs in a plethora of biological procedures associated both with health and disease has led to extended research on these biologically naturally derived platforms as API delivery platforms [75]. The utilization of EVs both as therapeutics and vehicles for drug delivery presents several advantages compared to conventional drug delivery systems like liposomes and other nanoparticles [75,76]. Firstly, due to their natural origin, EVs seem to have a more biocompatible profile [77,78]. Nevertheless, the diverse sources of EVs necessitate specific preclinical tests on each occasion to assess their immunogenicity [79]. Moreover, the complex mixture of molecules within EVs target different biochemical pathways, resulting in a synergistic therapeutic effect, more efficient than that produced by individual factors [61]. Applying EV technology in drug delivery could aid in the efficient transport of complex cargos to the CNS in cases of neurodegeneration [80].

In a pioneering study conducted by Alvarez-Erviti et al., they achieved more than 60% knockdown of the enzyme beta-secretase 1 in mice by the administration of small EVs and showed some promising results in the treatment of AD. The EVs in the mentioned study were derived from genetically modified dendritic cells. The dendritic cells were engineered to express an exosomal membrane protein (Lamp2b) fused to the neuron-specific rabies viral glycoprotein (RVG) peptide [50]. Their research paved the way for the use of EVs as drug delivery platforms. In a similar study, researchers showed that the intravenous administration of RVG-conjugated mesenchymal small EVs (MSC-RVG-Exo) in APP/PS1 mice resulted in higher brain-targeting ability and a better modulation of neuroinflammation. This led to improved cognitive function in the mice [51]. RVG targeting strategies have also been applied in PD. Izco et al. found decreased α-synuclein aggregation and a loss of dopaminergic neurons after the administration of RVG-small EVs that delivered shRNA minicircles [52]. Similarly, Ren et al. concluded that RVG-small EVs were capable of effectively delivering therapeutic aptamer molecules in the brain, eliminating the pathogenic mechanisms in PD in in vitro and in vivo models [53].

Neuroinflammation is among the pathologies for which the therapeutic effect of EVs has been evaluated. Yang and colleagues showed that naive small EVs isolated from macrophages can utilize integrin lymphocyte function-associated antigen 1 and intercellular adhesion molecule 1 (ICAM-1), as well as carbohydrate-binding C-type lectin receptors, to interact with the brain microvessel endothelial cells forming the BBB. The in vivo model showed a five-fold increased accumulation of a cargo protein (brain-derived neurotrophic factor, BDNF) in the brain, presumably due to the upregulation of ICAM-1 in the brain endothelial cells of the neuroinflammation model when compared to the non-inflammatory model [54]. In 2018, two independent research groups studied the immunomodulatory effect of small EVs derived from mesenchymal stem cells (MSCs) in the APP/PS1 transgenic mice AD model. Both groups showed that the administration of the small EVs led to the downregulation of pro-inflammatory factors (TNF-α and IL-1β) and the upregulation of anti-inflammatory cytokines, and thus reduced cognitive impairment [55,56]. Furthermore, the intravenous administration of small EVs isolated from adipose stem cells (ASCs) before experimental autoimmune encephalomyelitis (EAE) onset inhibited T-cell activation and infiltration into the BBB. However, no significant treatment effect was found in the later stages of the disease [57]. 

More recently, Giunti et al. pointed out the role of miRNAs, particularly miR-467f and miR-466q, in the immunomodulatory actions of small EVs. By priming the MSCs with IFN-γ, an overexpression of miRNAs was observed, capable of affecting the activation of pro-inflammatory microglia. The researchers conducted experiments in an EAE model, a well-established mouse model of multiple sclerosis, where treatment with small EVs from the IFN-γ-primed MSCs led to a decreased expression of pro-inflammatory markers in the spinal cord of SOD1G93A mice [58]. 

Another study on a chronic EAE model supports the finding that small EVs isolated from INF-γ-activated MSCs reduce pro-inflammatory cytokines and increase regulatory T cells (T_reg_). The EVs were distributed mainly in the areas of inflammation and their administration resulted in reduced degenerative inflammatory and demyelinating activities [59]. Furthermore, Li et al. found out that the administration of small EVs from bone marrow MSCs to EAE mice had a positive effect on the M1/M2 macrophage balance of the immune response. Specifically, the expression of M2-related cytokines—IL-10 and transforming growth factor (TGF)-β—increased while the presence of M1-related factors—TNF-α and IL-12—decreased at a significant level. The above led to a decrease in infiltrated systemic immune cells and demyelination actions when compared to EAE control animal subjects [59].

Currently, only one clinical trial has been registered (phase I/II) to evaluate the safety and efficacy of adipose MSC-derived exosomes after intranasal administration to AD-positive patients. Although the trial has been open since 2020, no results have been published yet [81].

Although scientists are working on developing EV-based therapeutic systems, challenges are abundant. EVs’ biological role, origin, and molecular composition make them, at least theoretically, well suited for therapeutic use on their own or as drug delivery vehicles. Their biochemical complexity and heterogeneity, however, are also primary concerns when trying to ensure the necessary consistency for any pharmaceutical product [82,83].

## 4. Recent Advances in the Development of Hybrid Liposomal–EV Nanoplatforms

**Figure 3:**
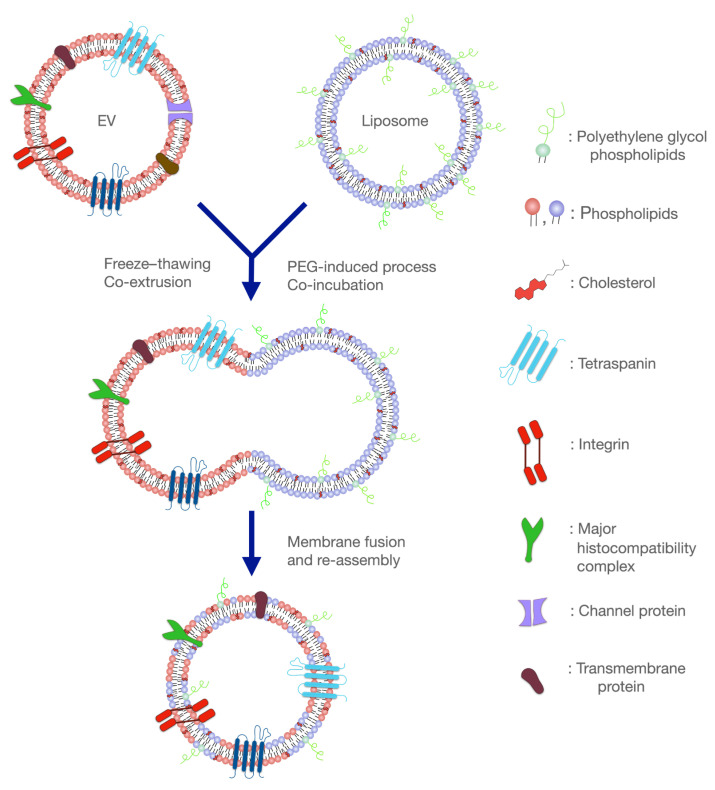
Fusion of liposomes and extracellular vesicles (EVs) for the development of the hybrid liposome–EV nanoplatforms. These systems combine the benefits of both delivery platforms and hold great promise in eliminating many diseases, including neurodegenerative disorders.

Recently, research has been conducted on the development of hybrid liposomal–EV nanoplatforms and their advantages as API delivery systems. Hybrid liposomal–EV nanoplatforms have the prospect of combining the advantages of each category (Figure 3).

On one hand, liposomes and lipid-based nanosystems in general are synthetic nanoparticles. Their development has been well defined and scale-up production approaches are available and applied by the pharmaceutical industry [84]. Different APIs, both hydrophilic and hydrophobic, have been successfully loaded and many innovative products are currently approved or under evaluation in terms of clinical practice [85]. However, due to their synthetic nature, they can easily trigger the immune response, thus decreasing their circulating time. Certain types of biomaterials in particular, such as cationic lipids and polymers, result in interaction with hydrophobic pockets of albumin, and finally the opsonization of the nanoplatforms [86]. Moreover, targeting specific areas can be challenging, especially in the case of delivering APIs in the brain, with the additional challenge of passing through the BBB. Nevertheless, even in the case when the targeting properties of lipid-based nanoparticles are efficient, endosomal escape is another big issue that these particles have to overcome [87].

On the other hand, EVs are biologically derived biocompatible nanoparticles. Their sheathing with a variety of trans-membrane proteins and other naturally derived factors helps them evade recognition by the immune system, allowing prolonged circulating time [88] and enhanced interaction with target cells [89,90]. Nevertheless, their complex cargo, which is not easily controlled, and low production and isolation yield limit their utilization in clinical practice for therapeutic purposes [61,91,92]. Consequently, the fusion of liposomal and EV membranes has led to the development of platforms with optimized delivery characteristics [93]. By fusing these two membrane types, higher drug loading flexibility is combined with the natural targeting properties of EVs [93]. 

The first hybrid system of that type was developed by Sato et al. in 2016, where they freeze–thawed liposomes together with small EVs to prepare their hybrid “bio-nanotransporters”. According to this study, hybrid EVs that consisted of PEGylated liposomes presented a higher cellular uptake when compared to those containing membranes of cationic liposomes. The researchers suppose that this might be due to the stereochemical-driven reduction in electrostatic repulsions between the hybrids’ anionic membrane parts and the cellular membranes or fusion properties of the PEG [94]. Another milestone study in this area was published by Piffoux et al. in 2018. The team formulated hybrid nanoplatforms by mixing MSC-derived small EVs and PEGylated liposomes. In this study, a PEG-driven approach was chosen to develop the vesicles instead of freeze–thawing cycles. Interestingly, the researchers could show that, when compared to commercially available anti-tumor liposomal dispersion, hybrid liposomal–EV nanoformulations presented an enhanced ability to deliver the drug molecules in 2D and 3D (spheroids) cell models of colon carcinoma [92]. These results are in accordance with the study of Mukherjee et al., who followed the same protocol. The mixing of EVs derived from an MCF-7 breast cancer cell line with cationic liposomes presented an increased siRNA delivery capacity in comparison to both the precursor EVs and liposomes with a 15% PEG ratio during the development process. However, the loading efficiency for the different types of hybrids, liposomes, and EVs has not been evaluated [95].

Cheng et al. proceeded to freeze–thaw thermosensitive liposomes with gene-engineered EVs that overexpressed CD47. The hybrid platforms were capable of delivering both a photothermal and an immune adjuvant agent simultaneously, with enhanced tumor accumulation. The combination of photothermal and immune therapy led to a significant elimination of tumor masses in mice models, paving the way for advanced therapies [96]. 

Another common process to develop hybrid EV–liposome nanoplatforms is extrusion through polycarbonate membranes of different pore sizes. Evers and coworkers compared the physicochemical characteristics, cell viability, and functionality of liposomes and hybrid nanoparticles. Interestingly, they found that although the cellular uptake was lower for the hybrid EV–liposome nanoplatforms in three different cell lines, their gene-silencing ability was maintained in the case of U87-MG cells. Thus, the authors suggest that maybe hybrids could achieve a better endosomal escape than liposomes. Finally, the MTS assay showed that hybrid EV–liposome nanoplatforms presented better biocompatibility than liposomes in the SKOV3 cell line [97]. 

The fusion of liposomes with cancer-derived EVs has been extensively reported. Jhan et al. studied the effect of different lipids in the development process of engineered EVs (eEVs). They observed that the lipids used in each case significantly affected the physicochemical characteristics (hydrodynamic diameter and ζ-potential) and the concentration of the eEVs. In contrast to the work by Piffoux mentioned above, in this case, a lower percentage incorporation of liposome membranes in the hybrid particles was achieved. For liposome–EV ratios 9:1, 4:1, and 1:1, non-important differences were observed in protein/lipid assays, indicating that each development method leads to different formulations of hybrid liposome–EV nanoplatforms [91]. Zhu et al. succeeded in achieving a high encapsulation efficiency of paclitaxel (~at 80%) in hybrids developed by mixing chimeric antigen receptor T-cell-derived exosomes with lung-targeting liposomes. In this way, the researchers achieved higher drug loading compared to the exosomes alone (~40%). The hybrid particles were capable of efficiently targeting the lungs due to the existence of the cationic lipids of liposomes and the release of paclitaxel to the tumors by active targeting through the mesothelin and PD-L1-specific single-chain variable fragments present in the exosomes [98]. 

Lin et al. managed to encapsulate large nucleic acid that included CRISPR/Cas9 into hybrid vesicles composed of liposomes and sEVs isolated by HEK293FT cells. The CRISPR/Cas9-based Runx2 gene expression regulation system proved to be more efficient for the hybrid vesicles when compared to sEVs, showing that these systems are capable of delivering larger APIs. However, the larger size and higher polydispersity index of the hybrid particles should be taken into consideration [99] as potential factors that could pose challenges in the in vivo application of these particles as drug delivery systems.

## 5. Concluding Remarks and Future Perspectives on Hybrid Liposome–EV Platforms in Neurodegeneration

In contrast to lipidic nanoparticles, especially for the well-established category of liposomes, both EVs and hybrid liposome–EV platforms present significant limitations in their translation to clinical practice. Low isolation yields, selection of the best parent cell type, and detailed evaluations of short- and long-term toxicity due to their complex cargoes are some of the main disadvantages when compared to lipidic nanoparticles. However, lipidic nanoparticles can still be improved to meet complex therapeutic needs. Thus, more elegant lipidic nanoparticles, which may target molecules such as peptides, are needed. The loading of lipidic nanoparticles with all these necessary factors is not an easy task, especially in terms of cost-efficient large-scale production. Indeed, although some preclinical studies have been performed utilizing lipidic nanoparticles for neurodegenerative disorders, their clinical evaluation is usually terminated in the early stages due to the inability to prove beneficial effects in disease progression, or without publishing the study results (Table 2). According to ClinicalTrials.gov [100], only one study in the treatment of neurodegenerative diseases resulted in beneficial outcomes for patients with transthyretin (TTR)-mediated amyloidosis (Table 2). Things do not seem different when we consider the limited research on EVs for the treatment of neurodegenerative disorders. First of all, the evaluation of their safety is crucial, as they may exhibit unexpected toxicity or side effects in clinical trials that are not observed in preclinical studies. Therefore, comprehensive toxicity studies are essential. Moreover, the development of reproducible manufacturing processes for both lipidic nanoparticles and EVs remains a challenge. Another significant challenge is related to the batch-to-batch variability of these platforms, which can limit their scale and adherence to good manufacturing practices.

**Figure 4:**
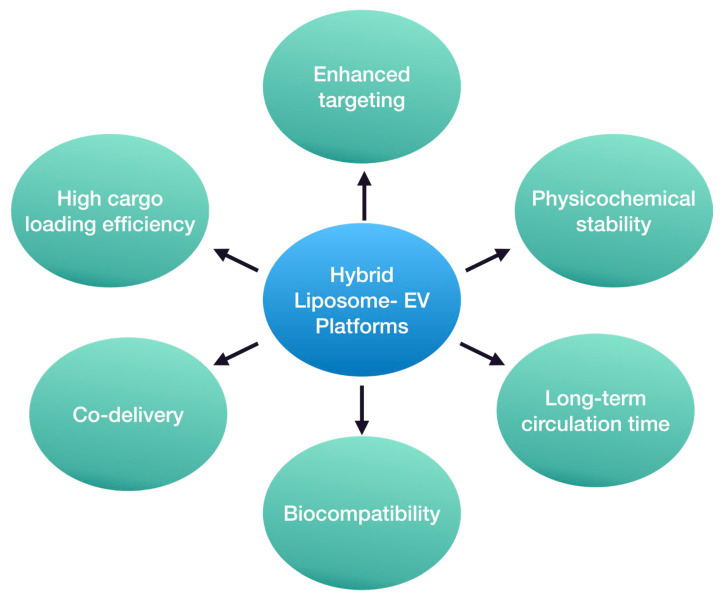
Potential advantages of hybrid liposome–EV platforms as API delivery nanosystems.

Undoubtedly, hybrid liposome–EV nanosystems hold great promise in the field of drug delivery, although much research remains to be conducted in this cutting-edge field (Figure 4). Current preliminary studies are focused on proof-of-concept developmental approaches or anti-tumor therapies. However, their potential role in the treatment of other diseases, such as neurodegenerative disorders, could be beneficial too. These disorders, including AD and PD, present significant challenges due to the complexity of the CNS [101,102]. Thus, the unique properties of hybrid liposome–EV nanosystems could offer advantages over conventional liposomes and EVs in overcoming some of the current obstacles by combining the beneficial key elements of both delivery platforms [103]. In this way, lipidic nanoparticles with enhanced loading abilities and patient-derived EVs with precise targeting capability could be used to provide personalized therapy and focus on the underlying causes of these disorders. Specifically, the utilization of EVs from neural cells to develop hybrid nanoparticles could facilitate API delivery and release to neural tissues. Consequently, the minimization of systemic adverse events and even a reduction in the required therapeutic dose could be achieved [104].

Moreover, these novel nanoplatforms might have a positive impact on the development of therapeutic approaches that focus on new pathways and molecular targets. For instance, the modulation of the immune response in the CNS and the regulation of neuroinflammation are currently being studied as promising alternatives to mono-therapeutic anti-amyloid and anti-TAU approaches [105,106]. Under this scope, hybrid liposome–EV nanoparticles could lead to high levels of API encapsulation efficiency and selective delivery to microglia and astrocytes. The combination of these characteristics is challenging without a delivery platform or with the use of liposomes or EVs alone. The use of hybrid liposome–EV platforms could aid in achieving this purpose.

One of the main benefits of hybrid liposome–EV platforms lies in their enhanced stability and biocompatibility. Neurodegenerative disorders often require prolonged and sustained drug delivery so that the API can effectively pass all the barriers, such as the BBB, and reach the desired tissue or target cells [103]. It is of high importance that APIs are delivered to the CNS in the therapeutic dose range, too. The lipid bilayer provides stability by protecting the cargo during circulation and transport through the BBB. At the same time, the endogenous origin of EVs can contribute to reduced immunogenicity and enhanced biocompatibility with the CNS environment.

Hybrid liposome–EV platforms allow great drug loading flexibility compared to the parent systems alone. Hybrid liposome–EV platforms can encapsulate or incorporate a broad range of therapeutic agents, including small drug molecules, proteins, and even larger nucleic acids. Moreover, these innovative platforms have proved to be efficient carriers of multiple agents [96]. Co-delivery could lead to promising results in the case of neurodegenerative disorders. For instance, in the case of AD, only symptomatic treatments have been clinically approved. Thus, an approach of combining the simultaneous administration of two therapeutic agents that target different pathophysiological routes could have a beneficial effect on disease progression. The co-delivery of genes and neuroprotective biomolecules through these platforms could also be used as a potential strategy to slow disease progression and promote the repair of damaged neural tissue [107].

A crucial issue that should be taken into account regarding novel API delivery platforms is the regulatory framework under which they should be evaluated. Lipidic nanoparticles, the only one of the three delivery representatives that are mentioned above and include authorized products in Europe and the U.S., follow the framework of non-biological complex drugs [108]. On the other hand, EVs are classified under the complex biological products category and their evaluation follows EMA/CAT/852602/2018 guidelines. Thus, a completely different approval procedure is followed by regulatory agencies, although both belong to the main group of advanced therapy medicinal products [109]. At a time when regulatory agencies are trying to establish the best parameters to categorize and evaluate liposomes and EVs, the addition of liposome–EV nanoformulations seems to make the process even more complicated. Ensuring the safety and efficacy of the platforms, as well as the development of consistent manufacturing practices, is necessary to obtain regulatory approvals. Collaboration between researchers and regulatory authorities is crucial to overcome these challenges and bring lipidic nanoparticles, EV-based therapies, or even hybrid liposomal–EV nanoplatforms to the clinic for neurodegenerative disorders.

In conclusion, there is great potential for utilizing hybrid liposome–EV nanoparticles in treating neurodegenerative disorders. Their enhanced stability, biocompatibility, flexibility in drug loading, targeted delivery, and potential for combining different APIs make them compelling candidates for the development of innovative treatments. These advantages observed in other diseases could also lead to a promising strategy to overcome challenges associated with drug delivery to the CNS and offer hope for more effective interventions in the realm of neurodegenerative diseases.

## Figures and Tables

**Table 2:** Clinical trials of liposomes and extracellular vesicles (EVs) according to ClinicalTrials.gov (February 2024).

Trial Number	Disease	Phase	Status	Sponsor
**EVs**				
NCT04202770	Refractory depression Anxiety disorders Neurodegenerative diseases	na	Suspended	Neurological Associates of West Los Angeles, Santa Monica, CA, USA
NCT04388982	AD	I/II	Unknown	Ruijin Hospital, Shanghai, China
**Liposomes**				
NCT04976127	PD	I	Active/not recruiting	InnoMedica Schweiz AG, Bern, Switzerland
NCT04654689	ALS	II	Completed	Fundación Universidad Católica de Valencia San Vicente Mártir, Valencia, Spain
NCT04601051	Transthyretin-related (ATTR) familial amyloid polyneuropathy Wild-type transthyretin cardiac amyloidosis Transthyretin-related (ATTR) familial amyloid cardiomyopathy	I	Active/not recruiting	Intellia Therapeutics, Cambridge, MA, USA
NCT01960348	Transthyretin (TTR)-mediated amyloidosis	III	Completed (with results)	Alnylam Pharmaceuticals, Cambridge, MA, USA

AD: Alzheimer’s disease; PD: Parkinson’s disease; ALS: amyotrophic lateral sclerosis; na: not applicable.
